# Supplementary material for: High proliferation and delamination during skin epidermal stratification
Source: Nat Commun. 2021 May 28;12:3227. doi: 10.1038/s41467-021-23386-4 (PMC8163813; doi:10.1038/s41467-021-23386-4)
Supplement: Supplementary file 1 — Supplementary Information [file 41467_2021_23386_MOESM1_ESM.pdf]

Supplementary Information

High proliferation and delamination during skin epidermal stratification

Damen et al., 2021

- **Supplementary Figures and Legends**
- **Supplementary Tables and Legends**

Supplementary Figure 1

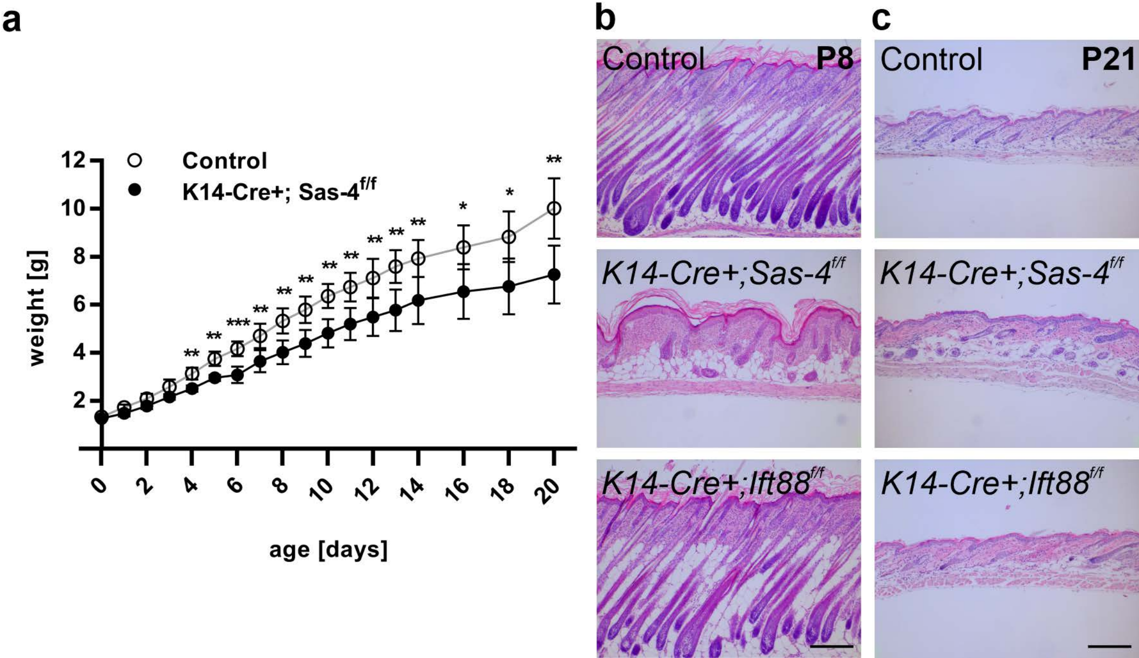

Supplementary Figure 1. Body weight and hair follicle defects upon centriole loss in stratified epithelia including the skin epidermis. (**a**) Measurements of the body weight of Control (n=7) and *K14-Cre+; Sas-4*$^{f/f}$ (n=6) mice from P0 to P20. * $p < 0.05$, ** $p < 0.01$, *** $p < 0.001$ (two-tailed student's T-test). Bars represent mean ± SD. (**b,c**) H&E histological staining of back-skin sections at P8 (**b**) and P21 (**c**) of Control, *K14-Cre+; Sas-4*$^{f/f}$ and *K14-Cre+; Ift88*$^{f/f}$ mice (scale bars: 100 μm).

Supplementary Figure 2

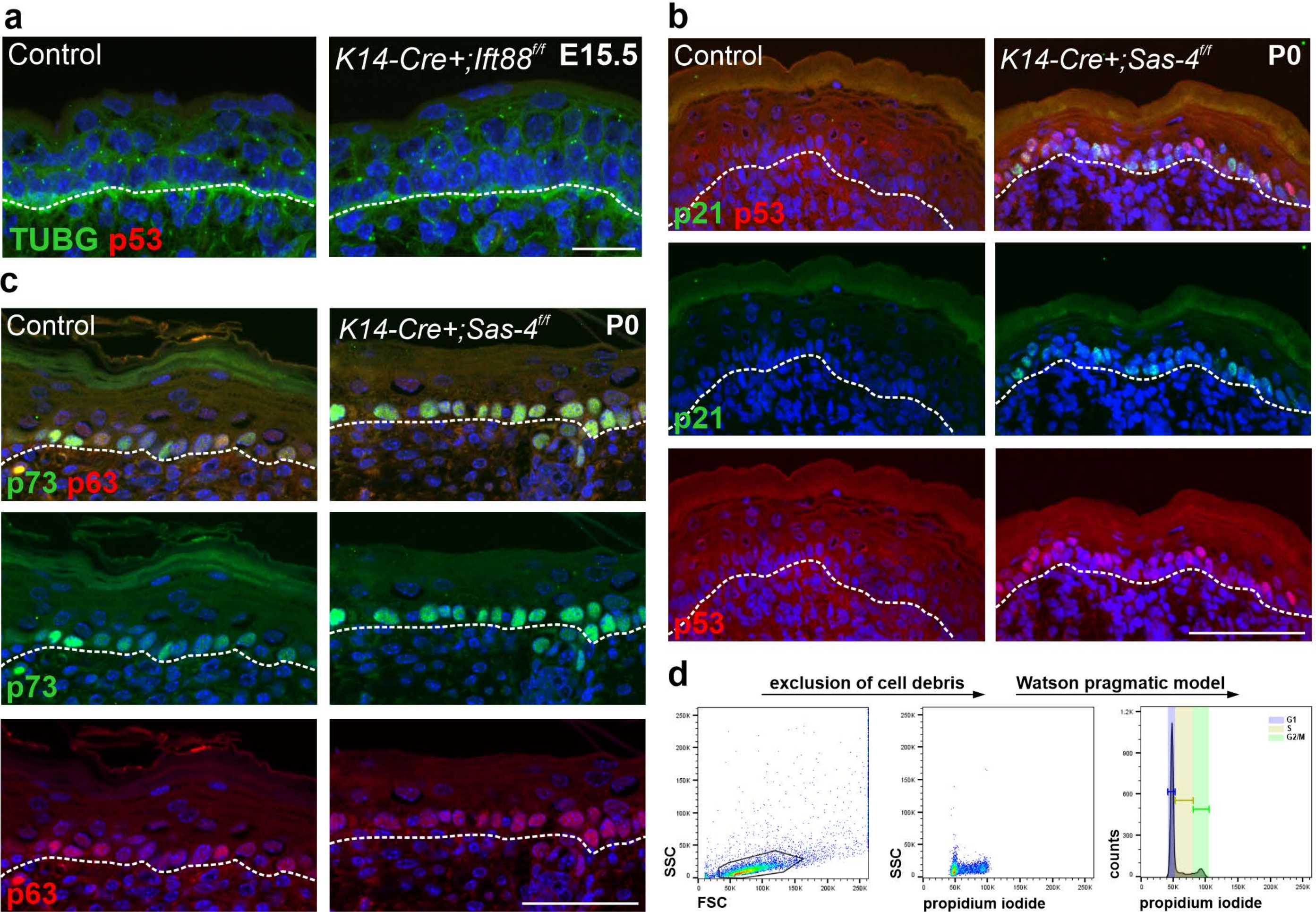

Supplementary Figure 2. p53 pathway in cilia and centrosome mutant epidermis. (**a**) Immunostaining of Control and *K14-Cre+; Ift88*$^{f/f}$ back-skin sections at E15.5 showing no detectable p53 upregulation (red) in cilia mutants (scale bar: 20 µm). TUBG (green) marks the centrosomes. Dashed line represents the epidermal-dermal interface in all panels. (**b,c**) Immunostaining of p21 (green) and p53 (red) (**b**) or p63 (red) and p73 (green) (**c**) of Control and *K14-Cre+; Sas-4*$^{f/f}$ back-skin sections at P0 (scale bars: 80 µm (**b**), 50 µm (**c**)). (**d**) Representative example of the gating strategy used for cell cycle analysis based on propidium iodide-stained keratinocytes from Controls shown in Fig. 2f. Cell debris were excluded by gating. The Watson pragmatic model was used to calculate the proportions of cells in G1, S and G2/M phase.

Supplementary Tables

Supplementary Table 1

Upregulated Genes			Downregulated Genes		
Ensembl ID	FoldChange	Gene	Ensembl ID	FoldChange	Gene
ENSMUSG00000034818	14.2	Celf5	ENSMUSG00000032011	-3.8	Thy1
ENSMUSG00000001986	13.1	Gria3	ENSMUSG00000010122	-3.1	Slc47a1
ENSMUSG00000046818	12.1	Ddit4l	ENSMUSG00000025348	-3.0	Itga7
ENSMUSG00000007783	12.1	Cpt1c	ENSMUSG00000020334	-2.7	Slc22a4
ENSMUSG00000024731	11.6	Ms4a10	ENSMUSG00000017737	-2.7	Mmp9
ENSMUSG00000026288	11.3	Inpp5d	ENSMUSG00000059049	-2.7	Frem1
ENSMUSG00000040350	11.1	Trim7	ENSMUSG00000091722	-2.6	Siah3
ENSMUSG00000075334	10.5	Rprm	ENSMUSG00000022754	-2.5	Tmem45a
ENSMUSG00000026399	9.5	Cd55	ENSMUSG00000048142	-2.5	Nat8l
ENSMUSG00000034457	9.2	Eda2r	ENSMUSG00000043441	-2.5	Gpr149
ENSMUSG00000097993	8.4		ENSMUSG00000030047	-2.5	Arhgap25
ENSMUSG00000073805	8.2	Fam196a	ENSMUSG00000096351	-2.4	Samd11
ENSMUSG00000040265	8.1	Dnm3	ENSMUSG00000033774	-2.4	Npbwr1
ENSMUSG00000031886	7.9	Ces2e	ENSMUSG00000073601	-2.3	Serpinb3c
ENSMUSG00000028610	7.6	Dmrtb1	ENSMUSG00000005125	-2.3	Ndrg1
ENSMUSG00000038295	7.4	Atg9b	ENSMUSG00000064128	-2.3	Cenpj
ENSMUSG00000028211	7.3	Trp53inp1	ENSMUSG00000050108	-2.3	Bpifc
ENSMUSG00000010651	7.3	Acaa1b	ENSMUSG00000068874	-2.2	Selenbp1
ENSMUSG00000074968	7.2	Ano3	ENSMUSG00000000305	-2.2	Cdh4
ENSMUSG00000037887	7.2	Dusp8	ENSMUSG00000034107	-2.2	Ano7
ENSMUSG00000048458	7.1	Fam212b	ENSMUSG00000041986	-2.2	Elmod1
ENSMUSG00000028600	6.7	Podn	ENSMUSG00000004328	-2.1	Hif3a
ENSMUSG00000044816	6.7	D630023F18Rik	ENSMUSG00000063626	-2.1	Unc5d
ENSMUSG00000037855	6.7	Zfp365	ENSMUSG00000017167	-2.1	Cntnap1
ENSMUSG00000028970	6.6	Abcb1b	ENSMUSG00000024743	-2.1	Syt7
ENSMUSG00000040432	6.5	Ltb4r2	ENSMUSG00000020695	-2.1	Mrc2
ENSMUSG00000021185	6.3	9030617O03Rik	ENSMUSG00000097050	-2.1	
ENSMUSG00000046908	6.3	Ltb4r1	ENSMUSG00000036908	-2.1	Unc93b1
ENSMUSG00000024778	6.2	Fas	ENSMUSG00000015970	-2.1	Chdh
ENSMUSG00000079022	6.0	Col22a1	ENSMUSG00000021779	-2.1	Thrb
ENSMUSG00000020467	5.6	Efemp1	ENSMUSG00000037579	-2.1	Kcnh3
ENSMUSG00000020326	5.6	Ccng1	ENSMUSG00000027750	-2.1	Postn
ENSMUSG00000002083	5.4	Bbc3	ENSMUSG00000036422	-2.1	Pcdh8
ENSMUSG00000042078	5.2	Svop	ENSMUSG00000107632	-2.1	
ENSMUSG00000023067	5.1	Cdkn1a	ENSMUSG00000028236	-2.1	Sdr16c5
ENSMUSG00000086245	5.1		ENSMUSG00000035112	-2.0	Wnk4
ENSMUSG00000019194	5.0	Scn1b	ENSMUSG00000032204	-2.0	Aqp9
ENSMUSG00000032135	5.0	Mcam	ENSMUSG00000046834	-2.0	Krt1
ENSMUSG00000036960	4.9	Clca2	ENSMUSG00000061928	-2.0	Dsg1b
ENSMUSG00000005107	4.8	Slc2a9	ENSMUSG00000026185	-2.0	Igfbp5

EnsemblID	FoldChange	Gene	EnsemblID	FoldChange	Gene
ENSMUSG00000058669	4.8	Nkx2-9	ENSMUSG00000096001	-2.0	2610528A11Rik
ENSMUSG00000044365	4.8	Cxxc4	ENSMUSG00000061517	-2.0	Sox21
ENSMUSG00000085389	4.8		ENSMUSG00000020027	-2.0	Socs2
ENSMUSG00000002020	4.8	Ltbp2	ENSMUSG00000111229	-2.0	
ENSMUSG00000042268	4.7	Slc26a9			
ENSMUSG00000059895	4.6	Ptp4a3			
ENSMUSG00000043557	4.4	Mdga1			
ENSMUSG00000068335	4.4	Dok1			
ENSMUSG00000038776	4.3	Ephx1			
ENSMUSG00000058740	4.2	Kcnt1			
ENSMUSG00000098470	4.1	C1rb			
ENSMUSG00000032839	4.1	Trpc1			
ENSMUSG00000026831	4.0	1700007K13Rik			
ENSMUSG00000033578	4.0	Tmem35a			
ENSMUSG00000036596	4.0	Cpz			
ENSMUSG00000039450	3.9	Dcxr			
ENSMUSG00000073418	3.9	C4b			
ENSMUSG00000009216	3.9	Fam163b			
ENSMUSG00000040918	3.8	Slc19a2			
ENSMUSG00000059013	3.7	Sh2d3c			
ENSMUSG00000031877	3.7	Ces2g			
ENSMUSG00000045658	3.7	Pid1			
ENSMUSG00000036480	3.7	Prss56			
ENSMUSG00000023000	3.6	Dhh			
ENSMUSG00000105039	3.6				
ENSMUSG00000032064	3.6	Dixdc1			
ENSMUSG00000032057	3.6	4833427G06Rik			
ENSMUSG00000046449	3.5	C77370			
ENSMUSG00000041670	3.5				
ENSMUSG00000051879	3.5	Krt71			
ENSMUSG00000008167	3.5	Fbxw9			
ENSMUSG00000026249	3.5	Serpine2			
ENSMUSG00000007908	3.5	Hmgcll1			
ENSMUSG00000034394	3.5	Lif			
ENSMUSG00000027663	3.4	Zmat3			
ENSMUSG00000086825	3.4				
ENSMUSG00000015451	3.4				
ENSMUSG00000109608	3.4				
ENSMUSG00000038963	3.4	Slco4a1			
ENSMUSG00000020423	3.4	Btg2			
ENSMUSG00000051811	3.4	Cox6b2			
ENSMUSG00000002257	3.3	Def6			
ENSMUSG00000095993	3.3				
ENSMUSG00000031026	3.3	Trim66			
ENSMUSG00000023032	3.3	Slc4a8			

EnsemblID	FoldChange	Gene	EnsemblID	FoldChange	Gene
ENSMUSG00000028195	3.3	Cyr61			
ENSMUSG00000041889	3.2	Shisa4			
ENSMUSG00000039813	3.1	Tbc1d2			
ENSMUSG00000033740	3.1	St18			
ENSMUSG00000027931	3.1	Npr1			
ENSMUSG00000020963	3.1	Tshr			
ENSMUSG00000031374	3.1	Zfp92			
ENSMUSG00000011263	3.0	Exoc3l2			
ENSMUSG00000028480	3.0	Glipr2			
ENSMUSG00000086040	3.0	Wipf3			
ENSMUSG00000021668	3.0	Polk			
ENSMUSG00000032128	3.0	Robo3			
ENSMUSG00000028893	2.9	Sesn2			
ENSMUSG00000024521	2.9	Pmaip1			
ENSMUSG00000001025	2.9	S100a6			

Supplementary Table 1. Differentially expressed genes in the centrosome mutant epidermis at E13.5. List of Top 100 up- and down-regulated differentially expressed genes with ≥ 2-fold changes and p-values < 0.05 (two-tailed student's T-test) based on RNA-Seq analysis of Control and *K14-Cre+; Sas-4*$^{f/f}$ epidermis at E13.5. The positive values are upregulated in the mutants.

Supplementary Table 2

Upregulated Genes			Downregulated Genes		
EnsemblID	FoldChange	Gene	EnsemblID	FoldChange	Gene
ENSMUSG00000056054	53.0	S100a8	ENSMUSG00000096257	-150.1	Ccer2
ENSMUSG00000058354	52.8	Krt6a	ENSMUSG00000009216	-92.6	Fam163b
ENSMUSG00000063975	39.7	Slco1a5	ENSMUSG00000009097	-61.7	Tbx1
ENSMUSG00000042157	32.9	Sprr2i	ENSMUSG00000064325	-61.0	Hhip
ENSMUSG00000058669	28.4	Nkx2-9	ENSMUSG00000004892	-54.3	Bcan
ENSMUSG00000102308	27.8		ENSMUSG00000038754	-51.2	Elovl3
ENSMUSG00000023041	27.2	Krt6b	ENSMUSG00000057604	-36.6	Lmcd1
ENSMUSG00000085183	24.8		ENSMUSG00000027869	-34.6	Hsd3b6
ENSMUSG00000064232	22.3	Gm5414	ENSMUSG00000040612	-34.1	Ildr2
ENSMUSG00000056071	22.2	S100a9	ENSMUSG00000048013	-33.6	Krt35
ENSMUSG00000026387	20.8	Sctr	ENSMUSG00000048450	-32.7	Msx1
ENSMUSG00000050063	20.2	Klk6	ENSMUSG00000020140	-31.1	Lgr5
ENSMUSG00000046354	19.3	Defb14	ENSMUSG00000049685	-30.4	Cyp2g1
ENSMUSG00000023000	19.3	Dhh	ENSMUSG00000046470	-28.0	Sox18
ENSMUSG00000087659	19.0		ENSMUSG00000033491	-27.8	Prss35
ENSMUSG00000046402	18.4	Rbp1	ENSMUSG00000070990	-27.0	Foxe1
ENSMUSG00000055030	18.4	Sprr2e	ENSMUSG00000066113	-25.5	Adamtsl1
ENSMUSG00000024810	17.9	Il33	ENSMUSG00000035775	-25.1	Krt20
ENSMUSG00000042212	17.3	Sprr2d	ENSMUSG00000053626	-24.8	Tll1
ENSMUSG00000104554	17.2		ENSMUSG00000031891	-24.8	Hsd11b2
ENSMUSG00000017817	16.8	Jph2	ENSMUSG00000035258	-23.0	Abi3bp
ENSMUSG00000109145	16.1		ENSMUSG00000055937	-22.4	Krt28
ENSMUSG00000017002	15.4	Slpi	ENSMUSG00000055421	-22.2	Pcdh9
ENSMUSG00000046259	14.3	Sprr2h	ENSMUSG00000018868	-22.0	Pnpla5
ENSMUSG00000062991	14.1	Nrg1	ENSMUSG00000047730	-21.5	Fcgbp
ENSMUSG00000025020	14.0	Slit1	ENSMUSG00000026185	-21.5	Igfbp5
ENSMUSG00000030268	14.0	Bcat1	ENSMUSG00000031375	-21.3	Bgn
ENSMUSG00000109392	13.6		ENSMUSG00000069805	-21.3	Fbp1
ENSMUSG00000027925	13.5		ENSMUSG00000041272	-21.2	Tox
ENSMUSG00000110542	13.1		ENSMUSG00000073043	-20.9	Atoh1
ENSMUSG00000029377	12.6	Ereg	ENSMUSG00000031757	-20.4	Mt4
ENSMUSG00000053797	12.6	Krt16	ENSMUSG00000040950	-19.4	Mgl2
ENSMUSG00000021214	12.2	Akr1c18	ENSMUSG00000000440	-19.3	Pparg
ENSMUSG00000101199	12.2		ENSMUSG00000050511	-19.3	Oprd1
ENSMUSG00000104155	12.0		ENSMUSG00000021994	-18.9	Wnt5a
ENSMUSG00000048455	11.9	Sprr1b	ENSMUSG00000015665	-18.7	Awat1
ENSMUSG00000042244	11.5	Pglyrp3	ENSMUSG00000053441	-18.6	Adamts19
ENSMUSG00000068859	11.5	Sp9	ENSMUSG00000068407	-18.6	Rnase12
ENSMUSG00000026420	11.4	Il24	ENSMUSG00000001804	-18.5	Dsg4
ENSMUSG00000079451	11.4	Tmprss11g	ENSMUSG00000029544	-18.4	Cabp1
ENSMUSG00000026700	11.3	Tnfsf4	ENSMUSG00000056222	-18.2	Spock1
ENSMUSG00000027520	11.2	Zdbf2	ENSMUSG00000052974	-18.2	Cyp2f2

EnsemblID	FoldChange	Gene	EnsemblID	FoldChange	Gene
ENSMUSG00000079018	11.1	Ly6c1	ENSMUSG00000033579	-18.1	Fa2h
ENSMUSG00000054958	11.1	Nt5c1a	ENSMUSG00000041730	-18.0	Prrxl1
ENSMUSG00000092500	11.0		ENSMUSG00000044288	-17.7	Cnr1
ENSMUSG00000030098	10.9	Grip2	ENSMUSG00000032017	-17.7	Grik4
ENSMUSG00000050092	10.8	Sprr2b	ENSMUSG00000026934	-17.4	Lhx3
ENSMUSG00000044216	10.7	Kcnj4	ENSMUSG00000056427	-17.1	Slit3
ENSMUSG00000032057	10.4	4833427G06Rik	ENSMUSG00000051504	-16.6	Siglech
ENSMUSG00000044430	10.3	Klk12	ENSMUSG00000018907	-16.4	Alox12e
ENSMUSG00000024379	10.3	Tslp	ENSMUSG00000017588	-16.2	Krt27
ENSMUSG00000050635	10.3	Sprr2f	ENSMUSG00000000247	-16.2	Lhx2
ENSMUSG00000110808	10.2		ENSMUSG00000020102	-16.1	Slc16a7
ENSMUSG00000062345	10.2	Serpinb2	ENSMUSG00000058897	-15.7	Col25a1
ENSMUSG00000045349	10.2	Sh2d5	ENSMUSG00000110417	-15.6	
ENSMUSG00000026249	9.9	Serpine2	ENSMUSG00000047330	-15.6	Kcne4
ENSMUSG00000099452	9.8		ENSMUSG00000050505	-15.6	Pcdh20
ENSMUSG00000020062	9.7	Slc5a8	ENSMUSG00000017897	-15.5	Eya2
ENSMUSG00000064246	9.6	Chil1	ENSMUSG00000027570	-15.5	Col9a3
ENSMUSG00000022483	9.5	Col2a1	ENSMUSG00000026888	-15.3	Grb14
ENSMUSG00000097636	9.4		ENSMUSG00000015787	-15.2	Abo
ENSMUSG00000030155	9.4	Clec2e	ENSMUSG00000034353	-15.2	Ramp1
ENSMUSG00000107350	9.1		ENSMUSG00000074575	-14.9	Kcng1
ENSMUSG00000029378	9.1	Areg	ENSMUSG00000078505	-14.9	Gm436
ENSMUSG00000046159	9.0	Chrm3	ENSMUSG00000028782	-14.8	Adgrb2
ENSMUSG00000049758	8.9	Olfr1318	ENSMUSG00000075570	-14.7	Krt26
ENSMUSG00000046694	8.9	Fam46b	ENSMUSG00000049556	-14.6	Lingo1
ENSMUSG00000027420	8.8	Bfsp1	ENSMUSG00000006269	-14.4	Atp6v1b1
ENSMUSG00000054215	8.8	Sprr2k	ENSMUSG00000028132	-14.4	Tmem56
ENSMUSG00000061144	8.8	Spink12	ENSMUSG00000042258	-14.4	Isl1
ENSMUSG00000037613	8.5	Tnfrsf23	ENSMUSG00000060560	-14.3	Ces4a
ENSMUSG00000020839	8.5	Tmigd1	ENSMUSG00000047641	-14.1	Krt87
ENSMUSG00000043472	8.5	Lce3d	ENSMUSG00000024366	-14.1	Gfra3
ENSMUSG00000044748	8.5	Defb1	ENSMUSG00000030303	-14.1	Far2
ENSMUSG00000042474	8.4	Fcmr	ENSMUSG00000043485	-14.0	Krt34
ENSMUSG00000059230	8.4	Defb4	ENSMUSG00000022371	-14.0	Col14a1
ENSMUSG00000068885	8.4	Lce3f	ENSMUSG00000024497	-13.9	Pou4f3
ENSMUSG00000075602	8.3	Ly6a	ENSMUSG00000033595	-13.8	Lgi3
ENSMUSG00000029161	8.3	Cgref1	ENSMUSG00000050377	-13.7	Il31ra
ENSMUSG00000036814	8.0	Slc6a20a	ENSMUSG00000074971	-13.4	Fibin
ENSMUSG00000050359	8.0	Sprr1a	ENSMUSG00000078507	-13.3	Aadacl3
ENSMUSG00000074743	8.0	Thbd	ENSMUSG00000073902	-13.3	
ENSMUSG00000035020	7.9	Epgn	ENSMUSG00000052821	-13.2	Cysltr1
ENSMUSG00000029019	7.9	Nppb	ENSMUSG00000021751	-13.2	Acox2
ENSMUSG00000028610	7.8	Dmrtb1	ENSMUSG00000074736	-13.2	Syndig1
ENSMUSG00000030790	7.6	Adm	ENSMUSG00000051251	-13.0	Nhlh1
ENSMUSG00000068349	7.5	Gml	ENSMUSG00000005836	-13.0	Gata6

EnsemblID	FoldChange	Gene	EnsemblID	FoldChange	Gene
ENSMUSG00000095385	7.5	D630033O11Rik	ENSMUSG00000050315	-13.0	Synpo2
ENSMUSG00000028970	7.3	Abcb1b	ENSMUSG00000007480	-12.7	Mc5r
ENSMUSG00000046203	7.2		ENSMUSG00000010505	-12.7	Myt1
ENSMUSG00000034275	7.1	Igsf9b	ENSMUSG00000026875	-12.6	Traf1
ENSMUSG00000032128	6.9	Robo3	ENSMUSG00000047992	-12.6	Fam69c
ENSMUSG00000032131	6.9	Abcg4	ENSMUSG00000028492	-12.5	Saxo1
ENSMUSG00000042425	6.9	Frmpd3	ENSMUSG00000047361	-12.5	Gm973
ENSMUSG00000043518	6.8	Rai2	ENSMUSG00000067615	-12.3	Krt81
ENSMUSG00000007783	6.8	Cpt1c	ENSMUSG00000070643	-12.1	Sox13
ENSMUSG00000016346	6.8	Kcnq2	ENSMUSG00000087383	-12.1	
ENSMUSG00000046449	6.7	C77370	ENSMUSG00000036242	-12.0	3632451O06Rik
ENSMUSG00000004885	6.7	Crabp2	ENSMUSG00000020913	-12.0	Krt24
ENSMUSG00000001986	6.7	Gria3	ENSMUSG00000044156	-11.9	Hepacam2

Supplementary Table 2. Differentially expressed genes in the centrosome mutant keratinocytes at P0. List of Top 100 up- and down-regulated differentially expressed genes with ≥ 2-fold changes and p-values < 0.05 (two-tailed student's T-test) based on RNA-Seq analysis of Control and *K14-Cre+; Sas-4*$^{f/f}$ epidermal keratinocytes at P0. The positive values are upregulated in the mutants.

Supplementary Figure 3

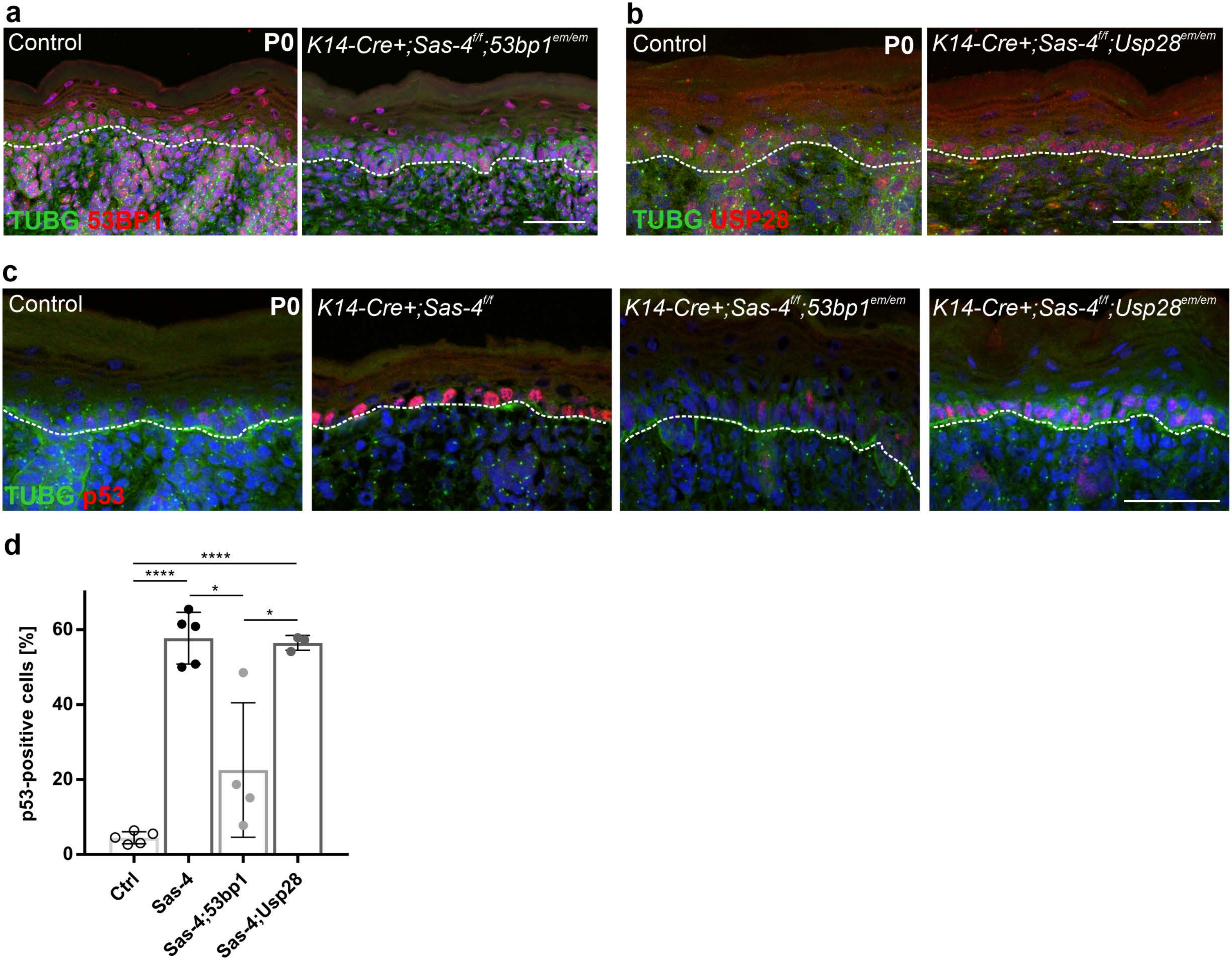

Supplementary Figure 3. The CRISPR/Cas9-generated hypomorphic alleles for *53bp1* and *Usp28* and their effect on p53 levels in centrosome mutants. (**a**,**b**) Immunostaining of back-skin sections at P0 of Control and *K14-Cre+; Sas-4*$^{f/f}$*; 53bp1*$^{em/em}$ (**a**) or *K14-Cre+; Sas-4*$^{f/f}$*; Usp28*$^{em/em}$ (**b**) showing residual signal of 53BP1 (red) (**a**) or USP28 (red) (**b**) suggesting that they are hypomorphic alleles (scale bars: 50 µm). (**a**,**b**,**c**) TUBG (green) marks the centrosomes. Dashed line represents the epidermal-dermal interface in all panels (**c**,**d**) Immunostaining (**c**) and quantification (**d**) of p53 (red) in back-skin sections at P0 of Control (n=5), *K14-Cre+; Sas-4*$^{f/f}$ (n=5), *K14-Cre+; Sas-4*$^{f/f}$*; 53bp1*$^{em/em}$ (n=4) and *K14-Cre+; Sas-4*$^{f/f}$*; Usp28*$^{em/em}$ (n=4) (scale bar: 50 µm). The Control and *K14-Cre+; Sas-4*$^{f/f}$ are part of the same experiment in Figure 2b. * $p < 0.05$, **** $p < 0.0001$ (two-tailed student's T-test). Bars represent mean ± SD.

Supplementary Figure 4

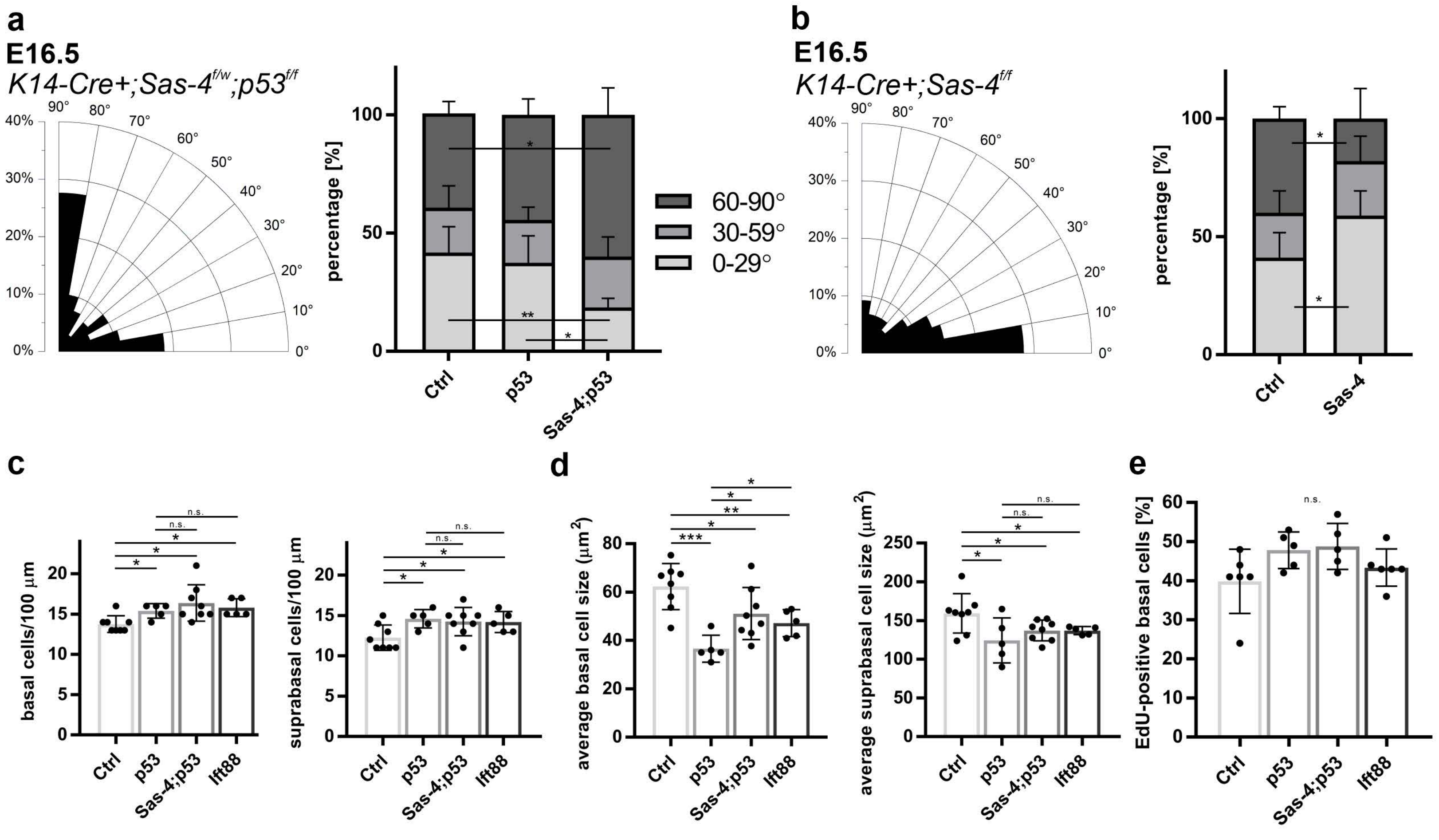

Supplementary Figure 4. Cell division orientation measurements, cell density and cell size in the epidermis of the different mutants and controls. (**a**,**b**) Radial histogram distribution of the angles of cell division in late anaphase to telophase of basal epidermal cells at E16.5 of *K14-Cre+; Sas-4*$^{f/w}$*; p53*$^{f/f}$ (**a**) and *K14-Cre+; Sas-4*$^{f/f}$ (**b**) animals. Percentages of parallel (0 - 29°), oblique (30 - 59°) and perpendicular (60 - 90°) divisions (right) of Controls (n=5), *K14-Cre+; Sas4*$^{f/w}$*; p53*$^{f/f}$ (n=3), *K14-Cre+; Sas-4*$^{f/f}$*; p53*$^{f/f}$ (n=5) (**a**), or Control (n=3) and *K14-Cre+; Sas-4*$^{f/f}$ (n=3) (**b**) animals. Control and *K14-Cre+; Sas-4*$^{f/f}$*; p53*$^{f/f}$ are parts of the same experiment shown in Fig. 4**c**. (**c**) Quantification of the density (cells per 100 µm) of basal and suprabasal layer cells of back-skin sections of the epidermis of Control (n=8), *K14-Cre+; Sas-4*$^{f/w}$*; p53*$^{f/f}$ (n=5), *K14-Cre+; Sas-4*$^{f/f}$*; p53*$^{f/f}$ (n=8) and *K14-Cre+; Ift88*$^{f/f}$ (n=5) mice at E16.5. Control and *K14-Cre+; Sas-4*$^{f/f}$*; p53*$^{f/f}$ are parts of the same experiment shown in Fig. 4**g**,**h**. (**d**) Quantification of the average cell size of basal and suprabasal layer cells in (**c**). Control and *K14-Cre+; Sas-4*$^{f/f}$*; p53*$^{f/f}$ are part of the same experiment shown in Fig. 4**i**,**j**. (**e**) Quantification of the percentage of the EdU-positive cells in the basal layer of back-skin epidermal sections of Control (n=6), *K14-Cre+; Sas-4*$^{f/w}$*; p53*$^{f/f}$ (n=5), *K14-Cre+; Sas-4*$^{f/f}$*; p53*$^{f/f}$ (n=5) and *K14-Cre+; Ift88*$^{f/f}$ (n=6) mice at E16.5. n. s. = not significant, * $p < 0.05$, ** $p < 0.01$, *** $p < 0.001$ (two-tailed student's T-test). Bars represent mean ± SD.

Supplementary Figure 5

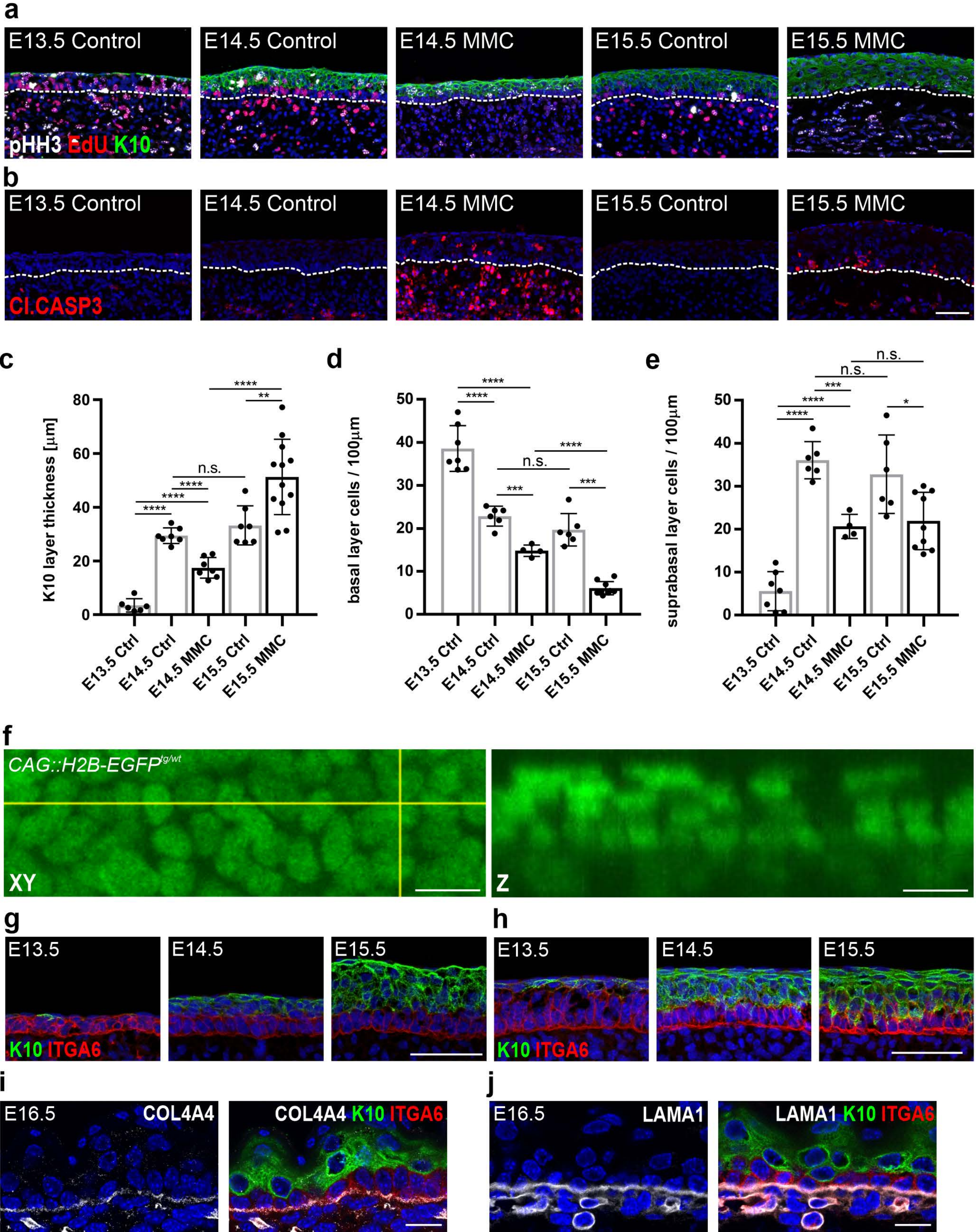

Supplementary Figure 5. Methods to culture, study and image skin explants. (**a**,**b**) Immunostaining for pHH3 (white), EdU (red), and K10 (green) (**a**) or Cl.CASP3 (red) (**b**) of Control and MMC-treated flat skin explants on filters at the indicated stages (scale bars: 50 µm). Dashed line represents the epidermal-dermal interface in all panels. (**c**) Quantification of the K10-positive layer thickness in the Control and MMC-treated skin explants. E13.5: Control n=6, E14.5: Control: n=7, MMC-treated: n=7, E15.5: Control: n=7, MMC-treated n=12 (**d**,**e**) Quantification of the density (cells per 100 µm) of basal (**d**) and suprabasal (**e**) layer cells of Control and MMC-treated skin explants. E13.5 Control: n=7, E14.5: Control: n=6, MMC-treated: n=4, E15.5: Control n=6, MMC-treated n=9) (**c,d,e**) n.s. = not significant, * $p < 0.05$, ** $p < 0.01$, *** $p < 0.001$, **** $p < 0.0001$ (two-tailed student's T-test or one-way ANOVA and Tukey's multiple comparisons test without adjustments). Bars represent mean ± SD. (**f**) Representative image of the CAG::H2B-EGFP (green) reporter taken from the time-lapses of flat skin explants at E14.5 shown in the XY (yellow lines) and Z dimensions (scale bars: 10 µm). (**g,h**) Immunostaining of the skin explant rolls (**g**), used for time-lapse imaging, and the flat skin explants (**h**) at E13.5-E15.5, for ITGA6 (red) and K10 (green) (scale bars: 50 µm). (**i,j**) Immunostaining of back-skin sections at E16.5 for the basement membrane marker COL4A4 (white) (**i**) or LAMA1 (white) (**j**) with ITGA6 (red) and K10 (green) (scale bars: 15 µm).

Supplementary Figure 6

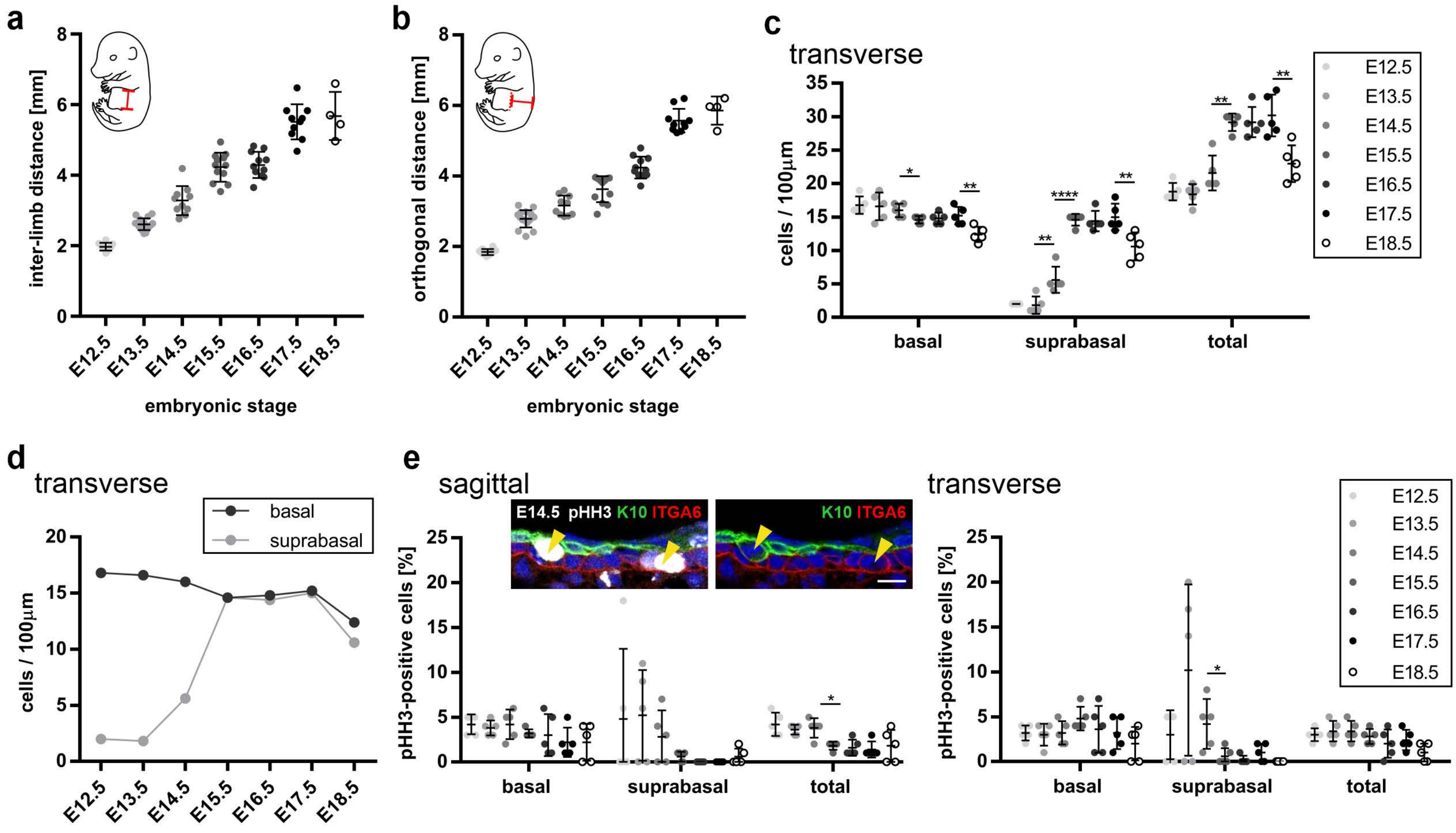

Supplementary Figure 6. Measurements of tissue growth, cell densities and mitotic indices in the different axes. (**a,b**) Quantification of the inter-limb distance (**a**) and orthogonal distance (**b**) as depicted in the embryo schematics (red lines) of wild-type embryos from E12.5-E18.5. E12.5: n=10, E13.5: n=16, E14.5: n=10, E15.5: n=12, E16.5: n=11, E17.5: n=10 and E18.5: n=4 (**c,d**) Cell densities (cells per 100 μm) of basal and suprabasal layer cells from transverse back-skin sections of the epidermis between E12.5-E18.5 (n = 5 independent animals). (**e**) Quantification of pHH3-positive (white) basal (ITGA6, red) and suprabasal (K10, green) cells of sagittal and transverse back-skin sections of the epidermis between E12.5-E18.5 (n = 5 independent animals), with examples of the immunostaining shown at E14.5 (arrowheads, scale bar: 10 μm). * $p < 0.05$, ** $p < 0.01$, **** $p < 0.0001$ (two-tailed student's T-test). Bars represent mean ± SD.

Supplementary Table 3

	53bp1	*Usp28*
Exon	Exon 2	Exon 2
gRNA	TACTGGAAGTCAATTGGATT	AATCAGCTGCGAGAAATCAC
Mutation	31 bp deletion	16 bp deletion
Deleted	GGACCCTACTGGAAGTCAATTGGATTCAGAT	CTGCGAGAAATCACAG
Primer 1	TCGAACTGATCTTTTGTTATTCCA	TCAAAAACAGAGCTGCCAGA
Primer 2	GAACAGGGCATCATCACTCA	CACACCTGACATGTGGGAAA

Supplementary Table 3. Endonuclease-mediated knockouts of *53bp1* and *Usp28*. List of used gRNAs and resulting mutation including used primer sequences.

Supplementary Table 4

Gene	Primer	Sequence
GFP	IMR 872 H2B GFP F	AAGTTCATCTGCACCACCG
GFP	IMR 873 H2B GFP R	TGCTCAGGTAGTGGTTGTCG
Cre	Cre_F	TGATGGACATGTTCAGGGATC
Cre	Cre_R	CAGCCACCAGCTTGCATGA
Trp53	Int10F	CACAAAAACAGGTTAAACCCA
Trp53	Int10R	GAAGACAGAAAAGGGGAGGG
Trp53	Rec1F	AAGGGGTATGAGGGACAAGG
Ift88	Ift88_wt	GCCTCCTGTTTCTTGACAACAGTG
Ift88	Ift88_fl	GGTCCTAACAAGTAAGCCCAGTGTT
Ift88	Ift88_delta	CTGCACCAGCCATTTCCTCTAAGTCATGTA
Sas-4	Cenpj LoxP2F:	TGCTTGCTTGTCTCTCCTGA
Sas-4	Cenpj LoxP2R:	GCTGACACCAAGTGGGAAAT
Sas-4	(delta) Cenpj FRT1F:	GGGGAGCAGACTTCAACACT
Usp28	Usp28Ex2short F2	TCAAAAACAGAGCTGCCAGA
Usp28	Usp28Ex2short R	CACACCTGACATGTGGGAAA
Trp53bp1	53bp1_Ex2 Fshort	TCGAACTGATCTTTTGTTATTCCA
Trp53bp1	53bp1_Ex2 Rshort	GAACAGGGCATCATCACTCA

Supplementary Table 4. Genotyping primers. List of used primers for genotyping.

Supplementary Table 5

Primary Antibodies					
Antigen	Species/ Isotype	Company	Catalog number	LOT number	Working dilution (IF)
53BP1	rabbit	Novus Biologicals	NB100-305SS	F-4	1:1000
ARL13B	rabbit	Proteintech	17711-1-AP	00023380	1:1000
CEP164	rabbit	Proteintech	22227-1-AP	00045133	1:2000
Cleaved-Caspase3	rabbit	Cell Signaling	9661	43	1:400
Collagen type IV	rabbit	Merck	AB756P	2909683	1:400
Integrin-α6	rat IgG2a	R&D Systems	MAB13501	INVV0117051	1:1000
Keratin1	rabbit	BioLegend	905601	D14BF00320	1:4000
Keratin10	guinea pig	Progen	GP-K10	911191	1:400
Keratin14	guinea pig	Progen	GP-CK14	603171	1:400
Keratin6A	rabbit	BioLegend	905701	B203935	1:4000
Ki67	rabbit	ThermoFisher	PA5-19462	GR3293888-1	1:400
Laminin	rabbit	Sigma Aldrich	L9393	125M4801V	1:1000
Loricrin	rabbit	BioLegend	905101	D15IF01641	1:2000
p21	mouse	Santa Cruz	sc-6246	B2715	1:50
p53 (CM5)	rabbit	Linaris	LIN-P956	6044554	1:1000
p63	mouse	Abcam	ab735	GR286167-1	1:25
p73	rabbit	Abcam	ab40658	EP436Y	1:1000
Phospho-Histone H3	rabbit	Merck	06-570	2724351	1:400
Survivin	rabbit	Cell Signaling	2808	15	1:400
USP28	rabbit	Sigma Aldrich	HPA006778	A105224	1:500
γ-Tubulin	mouse IgG1	Sigma Aldrich	T6557	049M4786V	1:2000

Secondary Antibodies					
Antigen	Species/ Isotype	Company	Catalog number	LOT number	Working dilution (IF)
AlexaFluor 488 α-mouse	goat	Invitrogen	A11029	1704587	1:1000
AlexaFluor 488 α-rabbit	goat	Invitrogen	A11034	2069632	1:1000
AlexaFluor 488 α-guinea pig	goat	Invitrogen	A11073	1737010	1:1000
AlexaFluor 568 α-mouse	goat	Invitrogen	A11031	1602786	1:1000
AlexaFluor 568 α-rabbit	goat	Invitrogen	A11036	2155282	1:1000
AlexaFluor 568 α-rat	goat	Invitrogen	A11077	1692966	1:1000
AlexaFluor 647 α-rabbit	goat	Invitrogen	A21245	1700082	1:1000
AlexaFluor 647 α-rat	goat	Invitrogen	A21247	1719171	1:1000
AlexaFluor 647 α-guinea pig	goat	Invitrogen	A21450	2026140	1:1000
Dyes					
DAPI	n.a.	AppliChem	A4099	n.a.	1:1000

Supplementary Table 5. Antibodies and dyes used for immunofluorescence stainings. List of primary antibodies, secondary antibodies, dyes, catalog numbers, LOT numbers and dilutions used for immunofluorescence stainings.
