# Supplementary material for: High proliferation and delamination during skin epidermal stratification
Source: Nat Commun. 2021 May 28;12:3227. doi: 10.1038/s41467-021-23386-4 (PMC8163813; doi:10.1038/s41467-021-23386-4)
Supplement: Supplementary file 3 — Description of Additional Supplementary Files [file 41467_2021_23386_MOESM3_ESM.pdf]

Description of Additional Supplementary Files

File Name: Supplementary Movie 1
Description: **An example of a perpendicular division orientation with the daughter cell pointing away from the basement membrane remaining within the basal layer.** Time-lapse imaging of the CAG::H2B-EGFP nuclear reporter in E13.5-E14.5 skin-roll explants with frames acquired every 7 minutes. The yellow dots highlight a perpendicular dividing basal cell and its daughter cells (scale bar: 10 µm). This example is representative of 44 out of 94 observed cells from 5 independent experiments (part of experiment shown in Fig. 5**a**).

File Name: Supplementary Movie 2
Description: **An example of a perpendicular division orientation with the daughter cell pointing away from the basement membrane lingering in a "second" layer.** Time-lapse imaging of the CAG::H2B-EGFP nuclear reporter in E13.5-E14.5 skin-roll explants with frames acquired every 7 minutes. The yellow dots highlight a perpendicular dividing basal cell and its daughter cells (scale bar: 10 µm). This example is representative of 50 out of 94 observed cells from 5 independent experiments (part of experiment shown in Fig. 5**b**).

File Name: Supplementary Movie 3
Description: **An example of a delaminating basal cell that divides in the suprabasal layers.** Time-lapse video of the CAG::H2B-EGFP nuclear reporter in E13.5-E14.5 skin-roll explants with frames acquired every 7 minutes shown in greyscale. The yellow dot highlights a basal progenitor which delaminates from the basal layer upwards and subsequently divides. The yellow dots mark the delaminating cell and its progeny (scale bar: 10 µm). This example is representative of a total of 46 cells from 5 independent experiments (part of experiment shown in Fig. 5**c**).
