# Supplementary material for: High proliferation and delamination during skin epidermal stratification
Source: Nat Commun. 2021 May 28;12:3227. doi: 10.1038/s41467-021-23386-4 (PMC8163813; doi:10.1038/s41467-021-23386-4)
Supplement: Supplementary file 7 — Reporting Summary [file 41467_2021_23386_MOESM7_ESM.pdf]

nature research

Corresponding author(s): Hisham Bazzi

Last updated by author(s): Apr 9, 2021

Reporting Summary

Nature Research wishes to improve the reproducibility of the work that we publish. This form provides structure for consistency and transparency in reporting. For further information on Nature Research policies, see our Editorial Policies and the Editorial Policy Checklist.

Statistics

For all statistical analyses, confirm that the following items are present in the figure legend, table legend, main text, or Methods section.

n/a	Confirmed	
☐	☒	The exact sample size (*n*) for each experimental group/condition, given as a discrete number and unit of measurement
☐	☒	A statement on whether measurements were taken from distinct samples or whether the same sample was measured repeatedly
☐	☒	The statistical test(s) used AND whether they are one- or two-sided<br>*Only common tests should be described solely by name; describe more complex techniques in the Methods section.*
☒	☐	A description of all covariates tested
☒	☐	A description of any assumptions or corrections, such as tests of normality and adjustment for multiple comparisons
☐	☒	A full description of the statistical parameters including central tendency (e.g. means) or other basic estimates (e.g. regression coefficient) AND variation (e.g. standard deviation) or associated estimates of uncertainty (e.g. confidence intervals)
☐	☒	For null hypothesis testing, the test statistic (e.g. *F*, *t*, *r*) with confidence intervals, effect sizes, degrees of freedom and *P* value noted<br>*Give P values as exact values whenever suitable.*
☒	☐	For Bayesian analysis, information on the choice of priors and Markov chain Monte Carlo settings
☒	☐	For hierarchical and complex designs, identification of the appropriate level for tests and full reporting of outcomes
☒	☐	Estimates of effect sizes (e.g. Cohen's *d*, Pearson's *r*), indicating how they were calculated

Our web collection on statistics for biologists contains articles on many of the points above.

Software and code

Policy information about availability of computer code

Data collection	Leica Application Suite X (LAS X, Leica, version 2.0.0.14332) and Zeiss ZEN lite (Zeiss, version 2.3) softwares
Data analysis	ImageScope (Leica, version 12.2.2.5015), ImageJ (NIH, version 1.53c), CellProfiler (Broad institute, version 3), OriginPro (Origin Lab, version 2019b), Prism 7 (GraphPad, version 7.03), Volocity (Improvision, version 6.3), Imaris (Bitplane, version 9.5.1), FlowJo (Tree Star, version 7.6.3), Molecular Signatures Database (GSEA, version v7.2), BioVenn (version 50), QuickNGS pipeline (version 1.2.4), Excel (Microsoft, Excel 2016)

For manuscripts utilizing custom algorithms or software that are central to the research but not yet described in published literature, software must be made available to editors and reviewers. We strongly encourage code deposition in a community repository (e.g. GitHub). See the Nature Research guidelines for submitting code & software for further information.

Data

Policy information about availability of data

All manuscripts must include a data availability statement. This statement should provide the following information, where applicable:

- Accession codes, unique identifiers, or web links for publicly available datasets
- A list of figures that have associated raw data
- A description of any restrictions on data availability

All the data that support the findings in this study are included within the paper and its supporting information. The RNAseq data has been deposited in the GEO repository under GSE161387 (https://www.ncbi.nlm.nih.gov/geo/query/acc.cgi?acc=GSE161387). Source data are provided with this paper.
The PID pathway database is accessible via the Molecular Signatures Database v7.2 of GSEA under http://www.gsea-msigdb.org/gsea/msigdb/genesets.jsp?collection=CP:PID.
The data that supports the findings of this study are provided in the main figures and supplementary figures, the published Source data file, or is available from the corresponding author upon reasonable request. The following figures have associated raw data:

Figure 1a,b,d-h, Figure 2a-j, Figure 3b-e, Figure 4a-j, Figure 5a-d, Figure 6a-e, Figure 7a, Suppl. Figure 1a-e, Suppl. Figure 1a-c; Suppl. Figure 2a-d; Suppl. Figure 3a-d; Suppl. Figure 4a-e; Suppl. Figure 5a-j; Suppl. Figure 6a-e.

Field-specific reporting

Please select the one below that is the best fit for your research. If you are not sure, read the appropriate sections before making your selection.

[x] Life sciences [ ] Behavioural & social sciences [ ] Ecological, evolutionary & environmental sciences

For a reference copy of the document with all sections, see nature.com/documents/nr-reporting-summary-flat.pdf

Life sciences study design

All studies must disclose on these points even when the disclosure is negative.

Sample size	No statistical method was used to predetermine sample size. The samples were independent biological replicates (animals) of n=3 or more and their number conformed to acceptable standards in the field and minimal statistical analyses requirements. Because our work involved animals, we employed the 3Rs to reduce the number of animals used in our experiments to the minimum requirements for statistical testing.
Data exclusions	No data obtained from technically successful experiments were excluded from the analyses.
Replication	The data were obtained from at least three independent experiments ensuring reproducibility.
Randomization	The samples were grouped by genotype or treatment and randomization was not applicable.
Blinding	The phenotype always correlated with the genotype and therefore blinding was not possible.

Reporting for specific materials, systems and methods

We require information from authors about some types of materials, experimental systems and methods used in many studies. Here, indicate whether each material, system or method listed is relevant to your study. If you are not sure if a list item applies to your research, read the appropriate section before selecting a response.

Materials & experimental systems

n/a	Involved in the study	
[ ]	[x]	Antibodies
[x]	[ ]	Eukaryotic cell lines
[x]	[ ]	Palaeontology and archaeology
[ ]	[x]	Animals and other organisms
[ ]	[x]	Human research participants
[x]	[ ]	Clinical data
[x]	[ ]	Dual use research of concern

Methods

n/a	Involved in the study	
[x]	[ ]	ChIP-seq
[ ]	[x]	Flow cytometry
[x]	[ ]	MRI-based neuroimaging

Antibodies

Antibodies used	The relevant information about antibodies is supplied in Supplementary Tables.
Validation	The antibodies used in this study were all commercial and validation information for application and antigen specificity is provided in the respective data sheets of the manufacturer.

Animals and other organisms

Policy information about studies involving animals; ARRIVE guidelines recommended for reporting animal research

Laboratory animals	Both male and female mice of C57Bl6/N and FVB/Nrj background were used in this study. Ages of the animals are indicated in the Figure legends. The animals were generated, housed and bred under standard conditions in the CECAD ivRF under a 12h light cycle, at a temperature of 22±2°C, 55±5% relative humidity and with food and water ad libitum.
Wild animals	No wild animals were used in this study.
Field-collected samples	No field-collected samples were used in this study.

Ethics oversight	The experiments involving animals were approved by the Landesamt für Natur, Umwelt, und Verbraucherschutz Nordrhein-Westfalen (LANUV), Germany (animal applications: 84-02.04.2014.A372, 84-02.04.2015.A405 and 81-02.04.2019.A476).

Note that full information on the approval of the study protocol must also be provided in the manuscript.

Human research participants

Policy information about studies involving human research participants

Population characteristics	Samples from both male and female individuals (controls and diagnosed with psoriasis or atopic dermatitis) within an age range between 5 and 79 years and of different body regions were provided by the Biobank of SFB829 Z4 platform.
Recruitment	Samples were obtained from the skin sample collection of the SFB829 Z4 Biobank.
Ethics oversight	The study protocol conformed to the ethical guidelines of the 1975 Declaration of Helsinki and was approved by the Ethics Committee of the Medical Faculty of University of Cologne (Registration No. 12-163). Informed consent has been obtained.

Note that full information on the approval of the study protocol must also be provided in the manuscript.

Flow Cytometry

Plots

Confirm that:

- [x] The axis labels state the marker and fluorochrome used (e.g. CD4-FITC).
- [x] The axis scales are clearly visible. Include numbers along axes only for bottom left plot of group (a 'group' is an analysis of identical markers).
- [x] All plots are contour plots with outliers or pseudocolor plots.
- [x] A numerical value for number of cells or percentage (with statistics) is provided.

Methodology

Sample preparation	Freshly isolated epidermal keratinocytes were fixed in 70 % ethanol and stored at -20 °C for several weeks. Then they were centrifuged at 77 x g, washed in 1x PBS and resuspended in Propidium Iodide staining solution (10 µg/ml PI, 200 µg/ml RNAse A, 0.1 % TritonTM X-100 in 1x PBS). After incubating at RT for 30 mins, cell cycle analysis was performed using a LSRFortessa (BD) FACS machine. Data were analyzed using the FlowJo software.
Instrument	LSRFortessa (BD)
Software	FlowJo software
Cell population abundance	All cells stained with PI were analyzed excluding cell debris.
Gating strategy	FSC/SSC gating was used to exclude cell debris. PI-stained cells were detected in PE channel and Watson pragmatic model implemented in FlowJo software was used to analyze the cell cycle proportions.

- [x] Tick this box to confirm that a figure exemplifying the gating strategy is provided in the Supplementary Information.
